# Wheat straw hydrochar induced negative priming effect on carbon decomposition in a coastal soil

**DOI:** 10.1002/imt2.134

**Published:** 2023-09-13

**Authors:** Xiao Wang, Zhen Li, Yadong Cheng, Hui Yao, Hui Li, Xiangwei You, Chengsheng Zhang, Yiqiang Li

**Affiliations:** ^1^ Marine Agriculture Research Center, Tobacco Research Institute Chinese Academy of Agricultural Sciences Qingdao China; ^2^ National Center of Technology Innovation for Comprehensive Utilization of Saline‐Alkali Land Dongying China; ^3^ Qingdao Key Laboratory of Coastal Saline‐alkali Land Resources Mining and Biological Breeding Tobacco Research Institute Qingdao China; ^4^ Department of Crop and Soil Sciences North Carolina State University Raleigh NC USA

## Abstract

The mechanisms underlying hydrochar‐regulated soil organic carbon (SOC) decomposition in the coastal salt‐affected soils were first investigated. Straw‐derived hydrochar (SHC)‐induced C‐transformation bacterial modulation and soil aggregation enhancement primarily accounted for negative priming effects. Modification of soil properties (e.g., decreased pH and increased C/N ratios) by straw‐derived pyrochar (SPC) was responsible for decreased SOC decomposition.

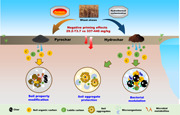

Progressive land‐use changes, deforestation, and the excessive combustion of fossil fuels have increased greenhouse gas (GHG) emissions and the widespread intensification of extreme weather events [1]. Global CO_2_ emissions have reached approximately 31.5 gigatons per year and are projected to triple by 2050 [2]. To address this issue, the Intergovernmental Panel on Climate Change (IPCC) appealed for GHG mitigation strategies [3]. As a typical representative of marginal soils, coastal salt‐affected soils, also referred to as blue C ecosystems [4], are beneficial for climate resilience and C sequestration [4]. However, in recent years, coastal soils have suffered from soil deterioration. Salt stress and nutrient deficiency caused the regression and degradation of soil primary productivity and substantial loss of blue C (0.15–1.02 Pg of CO_2_ released from the soil annually) from the coastal soils [5]. Therefore, reclaiming soil primary productivity is an urgent task to recover the ecological functions of these blue C ecosystems for climate change mitigation. Char amendment (e.g., pyrochar and hydrochar) as a soil C sequestration material has gained considerable attention for CO_2_ emission mitigation [6, 7]. Char amendment can increase, decrease, or have no effect on soil organic carbon (SOC) decomposition, corresponding to positive, negative, and no priming effect [8, 9]. Pyrochars, also known as biochars, are produced from the pyrolysis of dried biomass (e.g., straw wastes, sewage sludge, and animal manure) at 300–700°C [10]. Comparably, hydrochar produced from hydrothermal carbonization of wet biomass at lower temperatures (180–370°C) was an alternative method to pyrolysis for producing carbonaceous materials for soil C sequestration [11]. Given the considerable differences in thermal conversion and biomass conditions, the characteristics of hydrochars differ from those of pyrochars, which consequently affect their performance in CO_2_ emission mitigation [11, 12]. However, to date, most studies have focused on pyrochar effects on soil CO_2_ emission [13, 14] and limited attention has been paid to the corresponding effects of hydrochars.

Hydrochars have a low C sequestration potential for soils, mainly attributable to their high decomposability, and thus provide high‐level, easily degradable C and N sources for soil microbial activity [15]. Conversely, rice straw‐ and pig manure‐derived hydrochars decrease soil CO_2_ emissions due to the low bioavailability of inherent labile C and high C aromaticity [16]. Moreover, previous hydrochar studies have primarily focused on nonsalt‐affected soils, while data on salt‐affected soils remain limited. Compared with nonsalt‐affected soils, salt‐affected soils have low primary productivity and deteriorated physical structure, resulting in little input of exogenous organic matter [17] and weak protection of SOC by soil aggregates [18]. Corn straw‐derived pyrochars at 350°C and 550°C, characterized by high cation exchange capacity (CEC) and oxygen‐containing functional groups (e.g., –COOH and –OH), could decrease SOC decomposition (negative priming effect) mainly through promoting soil aggregation and shifting of bacterial community composition toward low C turnover bacteria in a coastal salt‐affected soil [19, 20]. The labile C component of char amendments can stimulate the secretion of functional cementing metabolites (e.g., proteins and organic acids) or residues by soil microorganisms to enhance soil aggregation, thereby decreasing SOC decomposition [21]. Unlike pyrochars, hydrochars with relatively higher amounts of labile C and N fractions could be more favorable for increasing microbial biomass abundance and shifting community composition and C‐cycling functions by altering soil organic matter (SOM) composition, substrate availability, and soil physicochemical properties (e.g., pH and enzyme activity), thus affecting SOC cycling [11]. It was reported that hydrochars could increase stable SOC fraction, mainly aromatic compounds, by decreasing the relative abundance of active bacterial decomposers of resistant SOC [22]. Additionally, the abundant O‐containing functional groups on hydrochars may enhance the soil aggregate stability to a greater extent than those on pyrochars by promoting the formation of char–organic matter–mineral complexes via hydrogen bonding and ligand exchange, thereby enhancing the physical protection of SOC by soil aggregates [19, 20]. However, the mechanisms of hydrochar‐mediated soil aggregation and microbially compositional and functional responses responsible for SOC decomposition in the coastal salt‐affected soils were poorly understood. To address this knowledge gap, a wheat straw‐derived hydrochar (SHC) produced at 220°C was prepared to investigate its effects on SOC decomposition from a coastal salt‐affected soil and the underlying microbial regulation and soil aggregation enhancement mechanisms in comparison with corresponding wheat straw‐derived pyrochar (SPC) pyrolyzed at 500°C using a 28‐day soil microcosm experiment; the objectives of this study are to: (1) compare the effects of SHC and SPC on SOC decomposition in coastal salt‐affected soil, (2) elucidate the mechanisms underlying char‐mediated soil aggregation and SOM composition associated with SOC decomposition, (3) identify the compositional and C metabolic responses of soil microbial communities to char amendments, and (4) elucidate the dominant factors determining char‐affected SOC decomposition.

## RESULTS AND DISCUSSION

### Char‐induced negative priming effect of SOC decomposition

**Figure 1:**
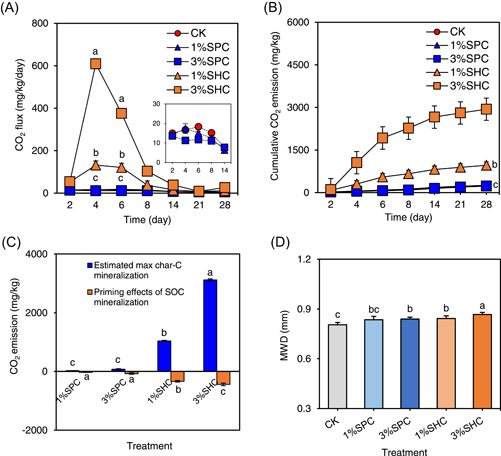
Effect of char amendments on the decomposition of soil organic carbon. CO_2_ flux (A), cumulative CO_2_ emission (B), estimated priming effects of soil organic carbon (SOC) decomposition (C), and calibrated aggregate stability index (D) in the coastal salt‐affected soils. Estimated maximum char‐C decomposition (CO_2_ emission) was calculated from the overall degradation amount of char labile C (dissolved organic carbon, Table S1). Priming effects of SOC decomposition were obtained by subtracting the estimated max char‐C decomposition from the total variation of CO_2_ emission between char treatment and CK treatment. Soil aggregate stability was indicated by values of mean weight diameter (MWD). The distribution patterns and MWD values of soil aggregates were calibrated by subtracting the size proportion and MWD values of straw‐derived pyrochar (SPC) and straw‐derived hydrochar (SHC) from the original experimental data (Figure S5). CK, soil without char amendment; SHC, straw‐derived hydrochar; SPC, straw‐derived pyrochar; 1%SPC and 3%SPC: soils amended with 1% and 3% (w/w) SPC; 1%SHC and 3%SHC: soils amended with 1% and 3% (w/w) SHC. The different lowercase letters represent significant difference between different treatments (Duncan's multiple‐comparison test, *p* < 0.05).

The CO_2_ fluxes in all soils generally increased with the prolonged incubation period, peaking on day 4, then gradually decreased in the later incubation period (Figure 1A). On day 4, SHC amendment at 1% and 3% markedly increased soil CO_2_ flux compared with that seen with soil without char amendment (CK) treatment, in the order of SHC at 3% > SHC at 1% (Figure 1A). Comparably, both SPC amendments at 1% and 3% exerted little effect on maximum soil CO_2_ fluxes relative to CK treatment. SHC amendment at 1% and 3% increased the cumulative soil CO_2_ emission by the end of the 28‐day incubation by 316% (959 mg/kg) and 1176% (2936 mg/kg) compared with CK, respectively (Figure 1B). The physicochemical properties of hydrochar and pyrochar were analyzed and are described in the Text S1. SHC contained a relatively higher dissolved organic carbon (DOC) content than SPC (104 vs. 2.42 mg/g, Table S1), which can act as labile C or bioavailable C fractions to be mineralized into CO_2_ by soil microbes, thereby contributing to the total cumulative CO_2_ emissions from soils amended with chars [14]. Consequently, the difference in DOC content between the SHC and SPC could affect their priming effects on SOC decomposition [14, 19]. Therefore, the net priming effects of SHC and SPC on SOC decomposition were calculated by subtracting the possible maximum C decomposition amount of SHC and SPC from the total detected cumulative CO_2_ emission [14] according to the DOC content of SHC and SPC (Figure 1B). For the SHC treatments, the increased CO_2_ emission (959–2936 mg/kg) induced by SHC amendments compared with that of CK treatment was lower than the total labile C amount of SHC (DOC, 1040–3120 mg/kg) (Table S1) added to the 1% and 3% SHC‐treated soils. This result suggested that SHC at 1% and 3% (w/w) could decrease SOC decomposition (negative priming effect) correspondingly up to 337 and 440 mg/kg (Figure 1C), respectively, in the coastal salt‐affected soils during a 28‐day incubation [14, 19]. Comparably, excluding the CO_2_ emitted from labile C degradation of SPC (DOC, 24.2–72.6 mg/kg) (Table S1) from overall CO_2_ emissions, SPC amendments at 1% and 3% induced negative priming effect up to 29.2 and 73.7 mg/kg, respectively (Figure 1C). This strongly agrees with our previous studies showing that pyrochar application to coastal salt‐affected soil resulted in a decrease in SOC decomposition [14, 19]. These results collectively confirm our hypothesis that SHC induces a greater negative priming effect on SOC decomposition in coastal salt‐affected soils than SPC. In the present study, the different effects of SOC decomposition induced by the SPC and SHC amendments could be mainly due to their different effects on soil physicochemical properties, including the distribution pattern and stability of soil aggregates, SOM availability, and microbial community responses. The different char‐induced SOC decomposition effects could be attributed to differences in char characteristics (Text S1). Relative to the SPC, the abundant carboxylic (–COOH) and hydroxyl (–OH) groups (Figure S1) in SHC could supply more adsorption sites for the labile C substrate, thereby resulting in a greater reduction in C availability for microbial utilization. However, the DOC contents in the SPC and SHC treatments were similar to that in the CK soil (Figure S2), excluding the direct sorption/immobilization of SOM by char amendments, which was the primary reason for the negative priming effect. However, compared with SPC, SHC with abundant O‐containing functional groups, could more efficiently enhance the stability of soil aggregates [19]. This may account for the greater SHC‐priming effect of SOC decomposition compared with that of SPC. Additionally, SPC and SHC can affect the diversity and composition of the soil microbial community and related enzymatic activities responsible for regulating soil C biochemical cycles and CO_2_ emission [22, 23].

Soil microbial metabolic activity is a key factor driving soil C cycling, especially SOC decomposition [24, 25]. As an important active component of soil C resources, microbial biomass carbon (MBC) is a sensitive indicator of soil process changes and contributes to the improved biological health of salt‐affected soils [26]. The microbial metabolic quotient is defined as the respiration rate per unit time of soil MBC and is generally used to measure microbial carbon use efficiency in soil [27]. Microbial C use efficiency (CUE) can indirectly affect SOC cycling by posing impacts on microbial biomass and necromass [28]. Accordingly, determining soil C‐transformation enzyme activity after char amendment is also necessary to better understand SOM decomposition and SOC decomposition [29]. More detailed results and discussion regarding the effects of SPC and SHC on the soil MBC, microbial metabolic quotient, and C‐transforming enzyme activity are presented in Text S2, Figures S3, S4.

### Enhanced stability of soil aggregates by char amendments

**Figure 2:**
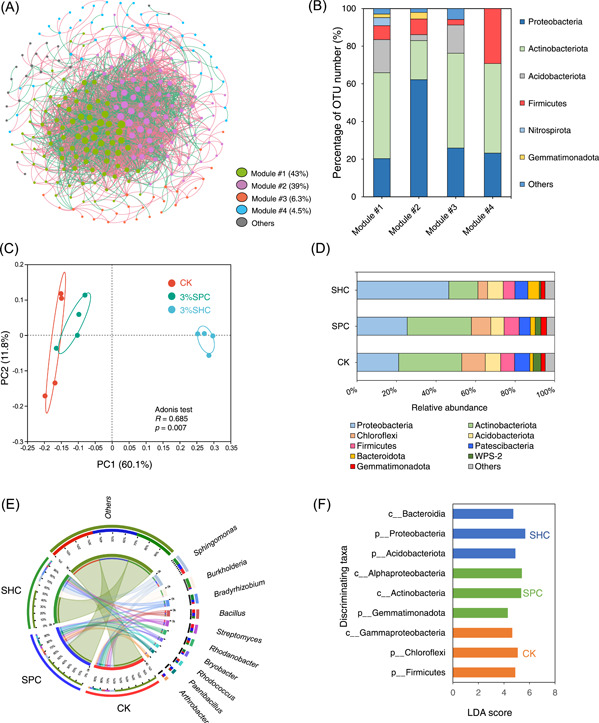
The bacterial community composition of soils with or without char amendments. Visualization of bacterial co‐occurrence network (A), percentage of operational taxonomic unit (OTU) number of the dominant bacterial phyla in the main ecological clusters (B), principal component analysis (PCA) based on the Bray–Curtis distance revealing the differences of soil bacterial community structure (C), the relative abundance of top 10 bacterial phyla (D), the relative abundance of 25 most abundant genera (E), and histogram of linear discriminant analysis (LDA) scores for discriminating bacterial phyla and classes between different treatments (F) (LDA score > 4.5). CK, soil without char amendment; SHC, straw‐derived hydrochar; SPC, straw‐derived pyrochar; SPC and SHC: soils amended with 3% (w/w) SPC and SHC, respectively.

The distribution pattern and stability of aggregates, a basic unit of soil structure, play critical roles in mediating SOC turnover and decomposition [30]. The char‐induced alternations in the proportion of soil macroaggregates, microaggregates, and MWD might have been attributed to the mechanical mixing of soil with the applied char, which passed through a 0.45‐μm [19, 31]. Due to the high stability (i.e., resistance to abiotic and biotic degradation) of char particles, the weight loss and size decrease of chars in soil aggregate fractions during incubation can be neglected [32, 33]. Therefore, the contribution of char particles to the aggregate proportion and MWD values was calibrated by subtracting the size proportion and MWD values of the SPC and SHC particles from the original experimental data (Figure S5). More detailed calibration methods are given in Text S3. The calibrated distribution patterns and stabilities of the soil aggregates are shown in Figure 2. For the macroaggregates (250–2000 μm) and microaggregates (53–250 μm), SPC and SHC additions increased their proportion compared with CK, following the order of 3%SHC > 1%SHC ≈ 3%SPC > 1%SPC (Figure S6C). Conversely, the proportion of silt–clay fractions (<53 μm) was substantially decreased after 3%SPC, 1%SHC, and 3%SHC amendments (Figure S6A,B), while SHC generally had a greater reduction effect than SPC (Figure S5C). These results demonstrated that SPC and SHC increase the macroaggregate and microaggregate amounts instead of the silt–clay fractions. As for the stability index of soil aggregates, the 3% SPC, 1% SHC, and 3% SHC additions significantly increased the MWD values by 4.26%, 4.67%, and 7.62%, respectively (Figure 1D). Comparably, the soil MWD values were slightly affected by 1% SPC, supporting our hypothesis that SHC posed greater promotional effects on soil aggregation than SPC. These results confirmed that the char‐elevated stability of soil aggregates could be attributed to the interactions between char and soil particles instead of the simple mechanical mixing between them.

Sporadic studies reported that pyrochar significantly promotes the formation and stability of aggregates in salt‐affected soils [32, 33]. Our previous study also demonstrated that corn pyrochars at 350°C and 550°C could enhance the stability of coastal salt‐affected soil aggregates resulting from the intimate physicochemical associations between SOM–mineral complex and pyrochar particles [19]. However, only a few studies have declared that pyrochar has little effect on salt‐affected soil aggregates [32, 34]. Char‐induced alterations in soil aggregation are largely affected by char attributes (application rate, feedstock type, and charring temperature), experimental conditions (land use and field duration), and soil properties (pH, physical texture, and initial SOC content) [35]. For the biochar attributes, the contents of oxygen‐containing functional groups (e.g., –COOH and –OH) and polyvalent cations (e.g., Ca^2+^ and Mg^2+^), which show high reactivity with soil minerals, mainly determine their effect on salt‐affected soil aggregation [8, 31]. Compared with SPC, SHC was enriched in O‐containing functional groups (Figure S1) and showed a greater probability of bridging with SOM and minerals to form organic matter–mineral complexes via hydrogen bonding and ligand exchange [31], which consequently showed stronger impacts on soil aggregation. This could explain the greater promotional effect of SHC than that of SPC on the formation of soil macroaggregates and microaggregates (Figure S6A,B). Additionally, char amendments can stimulate the secretion of microbial metabolites such as polysaccharides, amino acids, and organic acids by increasing the availability of soil nutrients and altering rhizosphere soil conditions, thereby increasing the stability of soil aggregates. As a result, the chars produced from mineral‐enriched feedstock (e.g., livestock manure and sludge) at a low temperature (<500°C) could have a more favorable influence on improving soil aggregation [32]. However, following char application, the responses of the microbial community and exudated metabolites largely vary, and the roles of char‐induced shifts in microbial community structure and metabolic functions and alterations of microbial metabolites in improving the structure and stability of soil aggregates in salt‐affected soils are poorly understood [36, 37]. In the present study, SHC contained relatively higher amounts of DOC and dissolved organic nitrogen (DON, 104 vs. 2.42 mg/g, 1.41 vs. 0.13 mg/g, Table S1) could more significantly stimulate the secretion of microbial metabolites through elevating the availability of soil nutrients (e.g., increasing total carbon (TC) and total nitrogen (TN) content in SHC‐treated soil than CK) (Figure S3B,C) than SPC, thereby increasing the stability of the soil aggregates [36, 37].

### Char‐altered response of bacterial community composition

Char amendment can change the microbial community structure and functional response resulting from its impact on substrate availability [20] and soil physicochemical properties (e.g., pH and exchangeable cation capacity) [38], thereby affecting microbially driven SOC decomposition processes. The Chao1, ACE, and Shannon indices were not affected by SPC or SHC amendments, but 3%SHC increased the Simpson index (Table S2), exhibiting the lifted bacterial community diversity following SHC amendments. Keystone ecological clusters of the network for all bacterial taxa at the phylum level were identified (Figure 2A). Four modules and three main network ecological clusters (module #1, 2, and 3) were detected in the bacterial co‐occurrence network, Actinobacteria was predominant in module #1, followed by Proteobacteria and Acidobacteria. Proteobacteria was dominant in module #2, followed by Actinobacteria. Actinobacteria was predominant in module #3, followed by Proteobacteria (Figure 2B). The phyla Actinobacteria, Proteobacteria, and Actinobacteria were the keystone nodes of typical ecological clusters. From the principal component analysis (PCA) analysis, SHC displayed different clustering features of the bacterial community compared with CK (Adonis test, *p* < 0.01). This suggests that the SHC treatment remarkably altered the composition of the soil bacterial community (Figure 2C). Comparably, SPC occupied a similar clustering feature of the bacterial community to that of the CK, implying little impact on the community composition posed by SPC.

The effect of char amendment on the bacterial abundance was further investigated (Figure 2D). SPC and SHC amendments increased the relative abundance of dominant bacterial node Proteobacteria by 20.1% and 119%, compared with CK, respectively (Figure 2D). Additionally, SHC increased the relative abundance of Acidobacteria, which are considered soil aggregation‐promoting and acid‐tolerant bacteria with high polysaccharide and enzyme secreion potential for transport and utilization of carbohydrates relative to CK [39, 40]. This implied that the enhanced soil aggregation in the SHC soils relative to CK (Figure 1D) could be driven by the bacterial responses associated with the transformation and secretion of these cementing agents. Similarly, the relative abundance of Bacteroidetes, copiotrophic bacteria with high turnover rates and activities in the C/N substrate plentiful soils [41], was remarkably increased after SHC amendment relative to CK, possibly resulting from the provision of higher‐level labile C substrates and other nutrients (NH_4_^+^‐N and TN content) in SHC‐applied soils than those of SPC (Figure S3). This was consistent with previous studies reporting that the pyrochar‐induced shift in microbial communities to copiotrophic taxa was driven by the increased availability of soil organic C substrates [42]. SHC generally increased the abundance of bacterial genera *Sphingomona* (ligninolysis bacteria) [40, 43], *Burkholderia* (cellulose hydrolysis bacteria, efficient decomposers of aromatic C) [44], and *Bryobacter* (active in aromatic hydrocarbon degradation) [45], showing an increasing trend for the degradation potential of polysaccharide‐like C substrate after SHC application, consistent with the increased content of humic‐like microbially degraded/transformed C products and proteins (C1 and C2) in the SHC‐treated soils (Figure S7, S8). The SPC had little influence on the abundance of these bacterial genera (Figure 2E). These differences in bacterial community composition between SPC and SHC were further confirmed by linear discriminant analysis (Figure 2F). At the phylum level, Gemmatimonadota was identified as a discriminating taxon for SPC amendment, while SHC treatment possessed discriminating bacterial phyla Acidobacteria and Proteobacteria. At the class level, *Actinobacteria* and *Alphaproteobacteria* were the discriminating bacterial taxa in response to SPC, whereas *Bacteroidia* was the discriminating bacterial class in response to SHC. SOM humification process was closely related to the microbial transformation of lignin‐like and condensed aromatic molecules [46]. Thus, the SHC‐triggered microbial function potential toward the efficient transformation of polysaccharide‐C/N substrates into highly reactive microbially derived carbohydrates and proteins could potentially promote soil humification and aggregation‐mediated soil SOC stabilization.

### Distinguished key factors determining the char‐affected SOC decomposition

**Figure 3:**
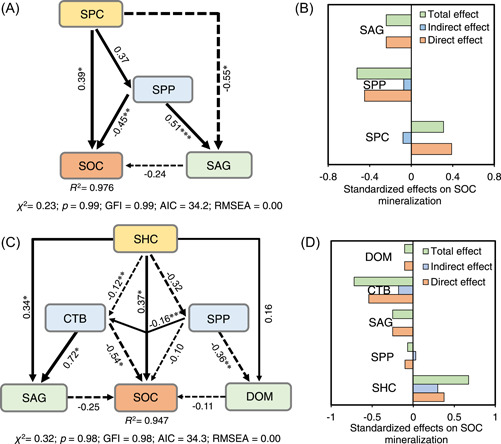
Structure equation model analysis evaluating the direct and indirect effects of key factors on the variations of SOC decomposition in the SPC (A, B) and SHC (C, D) amended coastal salt‐affected soils. Values on the arrows indicate path coefficient, and arrow width is proportional to path coefficients. Solid and dashed lines indicate positive and negative relationships, respectively. Significance levels are indicated by *(*p* < 0.05), **(*p* < 0.01), and ***(*p* < 0.001). The goodness of fit index (GFI > 0.90), chi‐square (*χ* ^*2*^ < 1), Akaike information criteria (AIC < 40), and root mean square errors of approximation (RMSEA is near 0) demonstrate that the data are well fitted with models. CTB, C‐transformation bacterial response; DOM, dissolved organic matter composition; PP, soil properties; SAG, soil aggregation; SPC, straw‐derived pyrochar; SHC, straw‐derived hydrochar.

The contributions of influential factors, such as char characteristics, bacterial responses, soil properties, aggregate stability, and DOM composition, to char‐induced alterations in SOC decomposition were evaluated by structural equation model (SEM) analysis (Figure 3). The physicochemical properties of SPC‐modified soil (decreased soil pH, increased C/N ratios, and TC content) were the greatest contributors to the reduction in SOC decomposition (Figure 3A,B). Comparably, SHC‐induced soil C‐transformation bacterial modulation predominantly contributed to decreased salt‐affected SOC decomposition (negative priming effects), followed by promoted soil aggregation and altered DOM composition (Figure 3C,D), clearly demonstrating the significant roles of SHC‐triggered bacterial modulation in affecting SOC decomposition, distinct from the SPC‐constructed models (Figure 3A,B). However, SHC characteristics (mainly pH and DOC content) were the greatest direct and positive contributors to decreased SOC decomposition, supported by the significant positive correlations between SHC and SOC. These results support our hypothesis that SHC induces the greater negative priming effect of SOC decomposition than SPC by shifting the microbial composition and promoting soil aggregation rather than the direct action of SHC itself as an exogenous C substrate in soils [19, 20]. Moreover, SHC‐promoted soil aggregation, one of the most important factors in SOC decomposition, was directly affected by the combined action of SHC and bacterial modulation of C transformation. This explains why the remarkably enhanced soil aggregation in the SHC soils was driven by the C‐transformation bacterial responses associated with increased SOM humification and generation of highly reactive metabolites (e.g., polysaccharides and organic acids) for the formation and stabilization of soil aggregates (Figure 1D). In addition, the SHC‐modulated C‐transformation bacterial response was significantly affected by soil properties (i.e., pH, soil C/N ratio, and TC content), which are conducive to SOC decomposition. This verified that SHC could induce bacterial responses involved in C transformation by altering soil conditions such as substance availability and salt stress, consistent with previous findings [22, 47]. Therefore, the key environmental factors shifting the composition of the soil bacterial community were further identified using RDA (Figure S9). The results showed that soil pH, regarded as a critical environmental factor regulating soil C cycles, microbial community composition, and metabolic potential [22, 47], mainly drove the shift in bacterial community composition. It was reported that hydrochars produced from poplar wood dust and wheat straw decreased the diversity of bacterial communities in paddy soils [15]. The authors attributed this to the fact that acidic soil conditions altered by hydrochars are not best suited for bacterial species that are favored under neutral conditions. However, in the present study, the acidic hydrochar (pH 4.15) (Table S1) lowered the soil pH from alkaline to neutral (Figure S3D). Therefore, the improved habitats for microbes by SHC may account for the shift in soil bacterial responses.

SHC characteristics (pH and DOC content) had a nonsignificant direct effect on soil dissolved organic matter (DOM) composition, implying that the alterations of DOM composition in SHC soils could not be primarily a result of the incorporation of the labile C component from SHC (Figure 3C). This was supported by the small effect of SHC on soil DOC content relative to CK (Figure S3A). Conversely, poplar wood dust and wheat SHC decreased the labile SOC fraction and increased the stable SOC fraction in paddy soil [22]. The authors ascribed the results to the hydrochar‐induced changes in the structural composition of bacterial communities, that is, reduced abundance of the condensed aromatic C degrader *Sphingobacterium* and increased abundance of bacterial decomposers of labile SOC. However, in the present study, the C‐transformation bacterial response did not contribute (non‐significant correlations) to soil DOM alterations, as revealed by the SEM models (Figure 3C). These differences could be explained by the different SOC stabilization pathways or statuses of the two tested soils. For instance, the short‐term promotional effects on microbial‐mediated C transformation after the application of hydrochar with high decomposability may not be apparent in clayed salt‐affected soils, where most C is chemically associated with minerals [48]. Collectively, the negative priming effects on SOC decomposition in the SHC‐amended soil were primarily driven by bacterial modulation and enhanced soil aggregation. Comparably, the modification of soil properties (e.g., decreased pH and increased C/N ratio) mainly accounted for the decrease in SOC decomposition in the SPC‐treated soils.

## CONCLUSION

The results demonstrated that SHC induced greater negative priming effects of SOC decomposition (35.2%–80.0% vs. 10.5%–31.5%) than those of SPC. SHC‐enhanced soil aggregate stability and humification process of SOM and increased abundance of bacterial taxa participated in the efficient transformation of condensed aromatic molecules into humic‐like substances were the underlying mechanisms. These findings provide novel insights into the potential roles of hydrochar in affecting the C biogeochemical cycle of salt‐affected soils and the basis for the development of robust measures to elevate the soil C sequestration potential of blue C ecosystems.

## AUTHOR CONTRIBUTIONS

Xiao Wang designed the research. Zhen Li performed the research. Yadong Cheng and Hui Yao conducted data analysis. Xiao Wang wrote and edited the manuscript. Hui Li, Xiangwei You, Chengsheng Zhang, and Yiqiang Li edited the manuscript. All authors have commented on and approved the final manuscript.

## Supporting information

Supporting information.

## Data Availability

New sequencing data was used in this article. The 16S rRNA gene amplicon was uploaded to NCBI under the accession number SRR25706658 (https://www.ncbi.nlm.nih.gov/sra/PRJNA1007573). Detailed information on the analysis process of bacterial 16S rRNA gene amplification [49] and co‐occurrence bacterial network construction [50] are given in Supporting Information. Supporting Information (figures, tables, scripts, graphical abstract, slides, videos, Chinese translated version, and update materials) may be found in the online DOI or iMeta Science http://www.imeta.science/.
