# Supplementary material for: Wheat straw hydrochar induced negative priming effect on carbon decomposition in a coastal soil
Source: Imeta. 2023 Sep 13;2(4):e134. doi: 10.1002/imt2.134 (PMC10989761; doi:10.1002/imt2.134)
Supplement: Supplementary file 1 — Supporting information. [file IMT2-2-e134-s001.docx]

***Supplementary Information***

**Wheat straw hydrochar induced negative priming effect on carbon decomposition in a coastal soil**

**Running title**: Hydrochar inhibited carbon decomposition

Xiao Wang^1,2,3^, Zhen Li^1^, Yadong Cheng^1,2,3^, Hui Yao^1,2,3^, Hui Li^4^, Xiangwei You^1,2,3,^*, Chengsheng Zhang^1,2,3^, Yiqiang Li^1,2,3,^*

^1^Marine Agriculture Research Center, Tobacco Research Institute, Chinese Academy of Agricultural Sciences, Qingdao 266101, China

^2^National Center of Technology Innovation for Comprehensive Utilization of Saline-Alkali Land, Dongying 257300, China

^3^Qingdao Key Laboratory of Coastal Saline-alkali Land Resources Mining and Biological Breeding, Qingdao 266101, China

^4^Department of Crop and Soil Sciences, North Carolina State University, Raleigh, NC 27695, United States

Correspondence: Xiangwei You and Yiqiang Li, Marine Agriculture Research Center, Tobacco Research Institute, Chinese Academy of Agricultural Sciences, Qingdao, China

Email: youxiangwei@caas.cn and [liyiqiang@caas.cn](mailto:liyiqiang@caas.cn)

**Table of Contents**

Text S1. Physic-chemical properties of hydrochar and pyrochar

Text S2. Char-shifted soil microbial C use efficiency and DOM compositions

Text S3. Methods

Table S1. Physic-chemical properties of soil and char amendments

Table S2. Alpha diversity indices of soil bacterial community

Figure S1. Characteristics of char amendments

Figure S2. Dynamics of coastal salt-affected soil properties

Figure S3. Microbial biomass carbon (MBC) and microbial metabolic quotient (qMB)

Figure S4. Enzyme activities of dehydrogenase, β-glucosidase, invertase and cellobiohydrolase of coastal salt-affected soils

Figure S5. Effect of char amendment on the original proportion of macroaggregates, microaggregates, silt-clay fractions and aggregate stability index of coastal salt-affected soils.

Figure S6. Effect of char amendment on the calibrated proportion of macroaggregates, microaggregates and silt-clay fractions of coastal salt-affected soils

Figure S7. Effect of char amendments on the composition of soil dissolved organic matter, including contour plots of four components and fluorescence spectra and intensity.

Figure S8. Three-dimensional fluorescence spectra of dissolved organic matter (DOM) extracted from control soil and char-treated soils

Figure S9. RDA analysis between bacterial community composition and soil properties

Figure S10. Spearman correlation analysis of soil CO_2_ emission, C-transforming enzyme activities and soil properties

**Text S1. Physic-chemical properties of hydrochar and pyrochar**

Characteristic results of SPC and SHC have been previously described [1,2]. Elemental analysis showed that bulk C content of SHC (53.2%) was significantly lower than that of SPC (57.6%), while SHC had higher TN content (1.12%) than SPC (0.94%). The atomic ratios of C/N and H/C of SHC were lower than SHC (Table S1), showing the relatively lower degree of carbonization and aromatization relative to SPC. Moreover, the higher contents of DOC, DON, and inorganic N (NO_3_^-^-N and NO_4_^+^-N) (Table S1) in SHC than SPC. The FTIR spectra exhibited that SHC had higher amount of O-containing functional groups such as -COOH, and aliphatic C-O/C-O-C, -OH (Figure S1) than SPC. Comparably, more aromatic C peaks, such as aromatic C-H and C=C/C=O, were observed in the spectra of SPC, indicating its higher aromaticity. Consistently, XPS spectra revealed the higher abundance of aromatic C (C-C/C=C/C-H) for SPC than SHC, which was agreed with more labile C constituents like DOC in SHC relative to SPC. Compared to SPC with stronger alkaline (pH 9.74), SHC had an acidic nature of pH 4.15 (Table S1) due to the presence of organic acids [3]. However, SHC possessed lower EC (0.52 dS/m) than that (0.72 dS/m) of SPC resulted from less concentrated minerals formed in hydrochar at the low HTT relative to pyrochar [3], explaining the higher ash content (12.5%) than that (25.3%) of SPC (Table S1).

**Text S2. Char-shifted soil** **microbial C use efficiency and DOM compositions**

Soil microbial metabolic activity is e a key factor driving soil C cycling, especially SOC decomposition [4,5]. As an important active component of soil C resources, microbial biomass carbon (MBC) is a sensitive indicator of soil process changes and contributes to the improved biological health of salt-affected soils [6]. In this study, 1%SHC and 3%SHC significantly elevated soil MBC content by 72.6% and 233%, respectively, compared with CK (Figure S3A). For SPC treatments, only adding 3% SPC significantly increased soil MBC content; 1% SPC did not pose similar effect, demonstrating that SHC could increase microbial community diversity and in turn elevate MBC content in salt-affected soils. The microbial metabolic quotient (qMB) is defined as the respiration rate per unit time of soil MBC and is generally used to measure microbial carbon use efficiency in soil [7]. Microbial C use efficiency (CUE) can indirectly affect SOC cycling by posing impacts on microbial biomass and necromass [8]. SPC at 3% (w/w) significantly decreased the qMB values in the soils compared with CK; the addition of 1% SPC had little effect on soil qMB values. The lower qMB, together with the increased MBC content in the SPC-treated soils (Figure S3B), indicated that more microbial biomass could be produced by soil microbes than rapid C turnover as SOC decomposition [9]. Comparably, SHC amendment increased qMB values (decreased microbial CUE) in the order of 3% SHC > 1% SHC (Figure S3B). This indicated that microbes allocated greater C to soil respiration or SOC decomposition, which lowered the conversion of C to MBC in the SHC treatments, despite the greater increase in MBC content induced by SHC than SPC (Figure S3A). Salt-affected soils characterized by high pH and salt content can induce osmotic stress to microorganisms, leading to the deterioration of habitats and reduction of nutrient substrates, thereby reducing the diversity of microorganisms [10,11]. Pyrochar has been reported to increase MBC and microbial C assimilation by enhancing the activities of C-acquiring enzymes (e.g., glucosidase, cellobiohydrolase, and dehydrogenase) in acidic soils; however, the effects were not significant in alkaline soils, which were limited by the low availability of SOM substrate [12]. The decomposition of SOM is mainly mediated by extracellular enzymes produced by microorganisms [13]. For instance, cellobiohydrolase degrades cellulose to smaller oligosaccharides, while glucosidase breaks down oligosaccharides into glucose [12,13]. Accordingly, determining soil C-transformation enzyme activity after char amendment is necessary to better understand SOM decomposition and SOC decomposition [12]. The effects of SPC and SHC on the C-transforming enzyme activity were investigated (Figure S4). The activity of dehydrogenase was significantly stimulated by 1% SHC and 3% SHC amendments relative to CK (Figure S4A); 1% SPC had a significant promotional effect on dehydrogenase activity, whereas 3% SPC had little influence on it. Similarly, 1% SHC and 3% SHC increased the glucosidase activity compared with CK, while SPC amendments at 1% and 3% had the dose-dependent decrease effect on glucosidase activity (Figure S4B). SHC at 1% and 3% correspondingly elevated them activities of invertase and cellobiohydrolase by 85.5–257%, relative to CK treatment (Figure S4C, D), showing the obvious promotional effect on C-acquiring enzyme activities by SHC compared with SPC. The differences in the char-affected microbial C use efficiency and enzyme activities between SHC and SPC could be ascribed to differences in the soil stoichiometric C:N ratio [14], soil conditions (e.g., soil aggregate structure, pH, and soluble salt content) [15] and microbial responses [16]. For instance, the triggered diversity and assimilation potential of the microbial community in the SPC treatments were not apparent where the majority of labile C substrates consisted of resistant and aromatic proteins and/or humic acid-like substances (Figure S7, S8) or did not significantly affect the soil pH after char amendment (Figure S3D). While for SHC addition, soil microorganisms may promote the SOM humification process by stimulating the breakdown of complex structural C macromolecules via invertase and cellobiohydrolase enzymes to acquire bound N and balance the soil stoichiometric C:N ratio [17]. Therefore, compared with SPC, SHC-elevated activities of C-transformation enzymes, a rate-limiting step in SOC decomposition, could facilitate the enhancement of soil C sequestration after char amendment. This was confirmed by the significant positive correlation between the activities of C-transformation enzymes (invertase, cellobiohydrolase, and glucosidase) and soil DOC content (Figure S10). The effects of char amendments on soil enzyme activity largely depend on the feedstock type, pyrolysis temperature for chars and variation in soil texture. Although in sandy and loamy soils, a meta-analysis study found that pyrochar produced at medium (350–550°C) and high (> 550°C) temperatures decreased the cellulase activity by 10.0% and 6.7%, respectively. By contrast, low temperature (< 350°C) pyrochar increased ligninase activity by 4.6–16.0% [17]. However, the role of hydrochar in regulating the activity of microbial C-transformation enzymes in alkaline salt-affected soils remains largely unknown. These results provide new evidence for the enzyme activities that affect the response of coastal salt-affected soil C sequestration to hydrochar.

DOM composition, which is most sensitive to the soil environment and is one of the important factors affecting soil C cycling [18,19] was investigated (Figure S7, S8). Four fluorescent components were identified (Figure S8A), including C1 (low molecular weight fulvic-acid substrates), C2 (phenolic, less aromatic structures and soluble microbial metabolic protein), C3 (humic acid-like substances consisting of phenols and other ingredients with aromatic structures), and C4 (aromatic protein containing tryptophan and tyrosine) [20,21]. The fluorescence spectra and maximum fluorescence intensities (*F*_max_) of the four SOM components are shown in Figure S8B. For C1, char amendments significantly elevated it compared with CK, following the order of 3% SHC > 3% SPC; 3% SHC increased the C2 content, while 3% SPC had little effect. However, 3% SPC amendment elevated the C3 and C4 contents, while 3% SHC decreased them. In the CK and SPC treatments, the soil DOM primarily consisted of C4, followed by C3 and C1 (Figure S8), whereas C1 (56.6%) and C2 (30.0%) occupied the dominant share of DOM components in the SHC-treated soils, showing the promoted humification process of SOM compared with SPC, supported by the results that hydrochar showed the great potential for humic acid production in soils [22]. This was consistent with the greater enhancement of soil aggregation in the SHC treatment than in the SPC treatment (Figure 1D), which was supported by the significant positive correlations between MWD values and C1/C2 content (Figure S10). The SHC-elevated MBC and qMB contents (Figure S3A, B) could be attributed to the supply of labile C substances (C1 and C2) by SHC for microbial growth and C assimilation and improved stresses (e.g., decreased soil pH and soluble salt content) of microhabitats [10,11,23]. This was confirmed by the significantly negative correlation between MBC/qMB and soil pH, and the positive correlation between MBC and labile C substance (e.g., DOC/C1/C2) content (Figure S10). However, converse results were reported that poplar wood dust and wheat straw derived hydrochars decreased the labile SOC fraction and increased stable SOC fraction (containing more aromatic C but fewer low molecular weight carbohydrate C) in neutral (pH 6.07–6.13) paddy soils [24]. These inconsistent results on hydrochar-induced alterations in SOM composition may be ascribed to differences in SOC composition and interactions of hydrochar with microbes between acidic paddy soils and alkaline salt-affected soils [2,25].

**Text S3. Methods**

**Text S3.1. Char amendments**

SHC is produced from the hydrothermal reaction (HTR) of wheat straw, a representative agricultural low-value solid waste. HTR was performed in a sealed 3 L autoclave (AC150, Shanghai, China) at a solid-to-liquid ratio of 1:10 (w/v), 220°C, and 50 MPa for 4 h [1]. The liquid-solid mixture was placed into a beaker and separated with a vacuum filtration apparatus (Tengjin, Shanghai, China) using a 0.5-μm polyethersulfone membrane; the resultant char products were oven dried at 60°C for 24 h for further experiment. Correspondingly, SPC is produced via slow pyrolysis at 500°C for 4 h using a box-type electric furnace (HDXQ-4-10, Hongda, China) under the N_2_ sweep gas [26] by ramping the temperature at a rate of 10ºC/min [27]. After charring, SHC and SPC were milled to pass a 0.45-μm sieve prior to the further experiment. The properties of SHC and SPC are presented in Table S1.

**Text S3.2. Microcosm experimental design**

In the present study, the treatments in triplicate were: (1) CK: soils without char amendments; (2) 1% SPC: soils amended with SPC at 1% (w/w); (3) 3% SPC: soils amended with SPC at 3% (w/w); (4) 1% SHC: soils amended with SHC at 1% (w/w); (5) 3% SHC: soils amended with SHC at 3% (w/w). Coastal salt-affected soil was sampled from the topsoil layer (0–20 cm) of an agricultural field in Dongying, China (118.67°N 37.42°E). The sampled soils were air-dried after removing plant residues and other debris, sieved to 2-mm and evenly homogenized. Then 300 g dried soil was packed into a 250-mL Erlenmeyer glass flask covered by sterilized aluminum films with 0.5 mm holes in a greenhouse at 25°C. The sterilized pure water was replenished into all the soils to reach 60% of maximum water holding capacity (WHC) for 7-day preincubation. SPC or SHC was then applied into the soils at 0%, 1% and 3% (w/w). All flasks were sealed with an airtight butyl rubber stopper with three-way valves, randomly arranged, and maintained using a light- and humidity-controlled greenhouse for 28 days. On days 2, 4, 6, 8, 14, 21, and 28, the efflux and cumulative emissions of CO_2_ from soils were determined using a gas chromatograph (Agilent 7890 B; Agilent Technologies Inc., USA) equipped with a flame ionization detector (Model 3800, Varian Inc., Walnut Creek, CA, USA) for CO_2_ analyses at 250°C. The CO_2_ emission flux (mg/g/day) and cumulative emission (mg/kg) were calculated [1,28]. For each CO_2_ efflux measurement, 50-mL gas in each flask was sampled. Subsequently, fresh air was pumped and flushed into the flasks for 5 min to supplement the air. Water loss was supplemented by the slow addition of deionized water. After 28-day incubation, fresh soils were analyzed for aggregate distribution and stability, microbial biomass C (MBC), C-transforming enzyme activity and bacterial community composition. MBC was measured using the [fumigation extraction](https://www.sciencedirect.com/topics/earth-and-planetary-sciences/fumigation-extraction) method [29]. Activity assays for invertase, glucosidase, cellobiohydrolase and dehydrogenase were conducted using a soil enzyme kit from Solarbio Science & Technology Co. (Beijing, China). Air-dried samples were prepared for analysis of pH, total C (TC), total nitrogen (TN), dissolved organic carbon (DOC), and organic nitrogen (DON) content.

**Text S3.3. Analysis of soil aggregate distribution and stability**

Considering coarse aggregate (> 2000 μm) was absent in the soils, three-sized soil aggregates, i.e., macroaggregate (250–2000 μm), microaggregate (53–250 μm), and silt-clay fraction (< 53 μm), were obtained from wet-sieving method [29]. Briefly, the 20 g fresh soil sample was submerged in the ultrapure water on the top of 2000 μm sieve for 3 min at 20 ± 1°C, and separated by moving the sieve up and down 50 times in 2 min. The aggregate fractions remaining on each sieve were air-dried for 48 h and weighed to obtain the dry mass. The particle size distribution characteristics of the soil aggregates were further analyzed and the mean weight diameter (MWD) of the soil aggregates was calculated as described in our previous study [29].

**Text S3.4. Analysis of soil dissolved organic matter**

The supernatant fraction was extracted from 2 g of fresh soil sample, and the fluorescence excitation-emission matrix (EEM) spectra of soil dissolved organic matter (DOM) were measured using a fluorescence spectrometer (F-4600, Hitachi, Japan) with a 150 W xenon arc lamp as the excitation source. Milli-Q water was used as a blank control, and a quartz cuvette with an optical path of 1 cm was used. EEM scans were performed at the excitation wavelengths ranging from 200 to 450 nm with a 5 nm scanning interval. The soil DOM fraction was divided into four components: C1, low molecular weight fulvic acid molecules; C2, phenolic, less aromatic structures, and soluble microbial metabolic proteins; C3, fulvic acid-like substances; C4, tryptophan protein. EEM-PARAFAC modeling for the EEM fluorescence data sets was established in MATLAB 2019 (Natick, MA, USA) using the DOM Fluor Toolbox [30,31]. The maximum fluorescence intensity (*F_max_*) of the individual components was used to indicate their relative abundance.

**Text S3.5. DNA extraction and high-throughput sequencing analyses**

Total DNA was extracted from fresh soil (0.5 g) using the PowerSoil DNA Isolation Kit (MO BIO Laboratories, Carlsbad, CA, USA). PCR amplification of the bacterial 16S rRNA gene in the V3–V4 hypervariable region was performed by the primers 338F (5’-barcode-ACTCCTACGGGAGGCAGCAG-3’) and 806R (5’-GGACTACHVGGGTWTCTAAT-3’) [26,32]. The PCR conditions, amplicon purification, and paired-end sequencing have been described in our previous study [26]. Sequence reads were processed and analyzed using QIIME (version 1.9.0) [33]. OTU taxonomic classifications were analyzed using RDP Classifier algorithm against the Greengenes database with a confidence threshold of 70%. The complexity of bacterial species diversity was analyzed using alpha diversity indices including the Chao1, ACE, and Shannon indices, using the QIIME (1.9.0). Chao1 and ACE were used to estimate bacterial community richness, whereas the Shannon index was used to estimate community diversity [33]. Principal component analysis (PCA) was conducted to visualize the dissimilarity in soil microbial diversity based on the Bray–Curtis dissimilarity matrix using the vegan package in R (version 3.1.2) [34]. Linear discriminant analysis Effect Size (LEfSe) was conducted to identify the discriminating taxa between the different treatments using the Meiji biological cloud platform (https://cloud.majorbio.com/page/tools/) [26]. Discriminating features were confirmed using *P* value of the factorial Kruskal-Wallis sum-rank test (*p* < 0.05) and the logarithmic linear discriminant analysis (LDA) score (> 3.5) [33,35]. Co-occurrence bacterial networks were constructed to visualize bacterial interactions. To avoid spurious correlations between OTUs, the Sparse Correlations for Compositional data (SparCC) method was used to construct bacterial co-occurrence network in R using the SPIEC-EASI package [33,35]. Only OTUs detected in at least 75% of samples were retained, and the final co-occurrence network only displayed statistically robust correlations (SparCC correlation coefficient, *r* > 0.7; significance level, *p* < 0.01) [35]. After network construction, network visualization and editing were performed using Gephi (<https://gephi.org/>). The topological role of each bacterial node was determined using within-module connectivity (*Zi*) and among-module connectivity (*Pi*). Network hubs (*Zi* > 2.5 and *Pi* > 0.62), module hubs (*Zi* > 2.5 and *Pi* < 0.62), and connectors (*Zi* < 2.5 and *Pi* > 0.62) were classified as keystone taxa [35].

**Text S3.6. Statistical analysis**

Significant differences between different treatments were analyzed using SPSS 22 (SPSS Inc., Chicago, USA) using one-way analysis of variance with Duncan’s multiple range test (*p* = 0.05), and Spearman’s correlation analysis at *p* = 0.05 was conducted using R software 4.0.3. Redundancy analysis (RDA) was used to identify the main factors affecting alterations in microbial community composition was performed by Canoco 5.0. Structural equation modeling (SEM) was conducted to estimate the factor contributions to variations in soil CO_2_ emissions using the R package vegan (piecewiseSEM). The normalized chi-square (≤ 3), goodness of fit index (> 0.9) and root mean square errors of approximation (< 0.08) reflected a good fitting of the SEMs [26].

**Table S1.** Physic-chemical properties of soil and char amendments.

| Soil^α^ | | Char^β^ | | |
| --- | --- | --- | --- | --- |
|  |  |  | SPC | SHC |
| pH | 7.98 | pH | 9.74b^γ^ | 4.15a |
| SOM (g/kg) | 26.1 | TC (%) | 57.6b | 53.2a |
| EC (dS/m) | 0.72 | TN (%) | 1.45a | 1.56b |
| AN (mg/kg) | 285 | C/N | 66.6b | 52.9a |
| AP (mg/kg) | 185 | H/C | 0.04a | 0.10b |
| AK (mg/kg) | 156 | Ash content (%) | 25.3b | 12.5a |
| CEC (cmol/kg) | 14.0 | NH_4_^+^-N (mg/kg) | 3.06a | 14.2b |
| WHC (%) | 53.1 | NO_3_^-^N (mg/kg) | 0.60a | 218b |
|  |  | DON (mg/g) | 0.13a | 1.41b |
|  |  | DOC (mg/g) | 2.42a | 104b |

^α^SOM: soil organic matter; AN: available nitrogen; AP: available phosphorus; AK: available potassium; CEC: cation exchange capacity; WHC: water holding capacity.

^β^SPC: wheat straw pydrochar; SHC: wheat straw hydrochar. Their properties were previously reported [1].

^γ^The different lowercase letters represent significant difference between different chars (Duncan’s multiple-comparison test, *p* < 0.05).

**Table S2.** Alpha diversity indices of soil bacterial community

| Treatments | Chao1^α^ | ACE | Simpson | Shannon |
| --- | --- | --- | --- | --- |
| CK | 2984 ± 58.3 a | 375 ± 2.84 a | 0.02 ± 0.00 a | 6.47 ± 0.06 a |
| 3%SPC | 3013 ± 17.2 a | 375 ± 3.96 a | 0.02 ± 0.00 a | 6.40 ± 0.09 a |
| 3%SHC | 2361 ± 167 a | 361 ± 10.1 a | 0.05 ± 0.00 b | 6.38 ± 0.18 a |

^α^ Chao1 and ACE indexes: the richness index of bacterial community; Simpson and Shannon indexes: the diversity index of bacterial community.

^β^ Different small letters after the data in the same row indicate signiﬁcant difference among different chars, which was analyzed by the Duncan’s multiple range test (*n* = 3, *p* < 0.05).


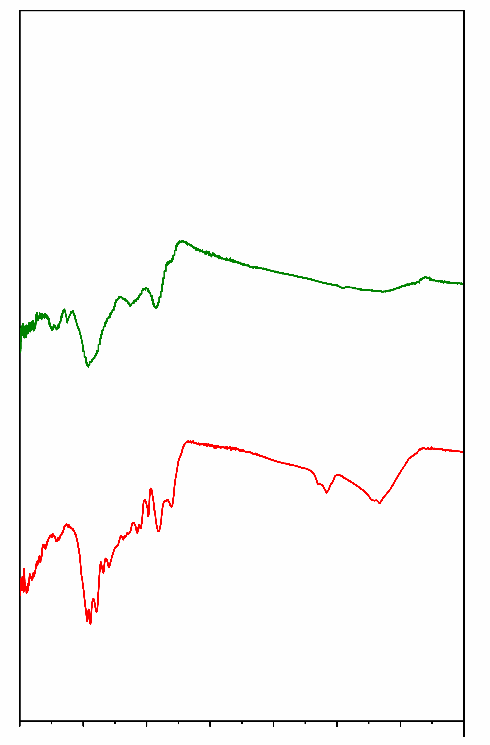


**(B)**

**(A)**

C=O

Aromatic

COOH

C=C/C=O

Aromatic

Aliphatic

**Wavenumber (nm)**

4000

3500

3000

2500

C-O/C-O-C

C-H

Aromatic

500

1000

1500

2000

Aliphatic

CH_2_

-OH

**SPC**

**SHC**

**Figure S1.** Characteristics of char amendments. FTIR spectra of (A) wheat straw derived pyrochar (SPC) and hydrochar (SHC), XPS carbon 1s spectra of SPC (B) and SHC (C). These results have been reported by our previous study [1].

**Figure S2.** Dynamics of coastal salt-affected soil properties. DOC (A), TC (B), TN (C), pH (D), C/N ratio (E) and DON (F) in the coastal salt-affected soils without or with char amendments. CK: soil without biochar amendment; 1%SPC and 3%SPC: soils amended with 1% and 3% (w/w) SPC; 1%SHC and 3%SHC: soils amended with 1% and 3% (w/w) SHC.

**Figure S3.** Microbial biomass carbon (MBC) (A) and microbial metabolic quotient (qMB) (B) in the coastal salt-affected soils. Soil aggregate stability was indicated by values of mean weight diameter (MWD). The distribution patterns and MWD values of soil aggregates was calibrated by subtracting the size proportion and MWD values of SPC and SHC from the original experimental data (Figure S5). The values of qMB were calculated as follows: qMB = SOC decomposition rate/MBC. CK: soil without char amendment; 1%SPC and 3%SPC: soils amended with 1% and 3% (w/w) SPC; 1%SHC and 3%SHC: soils amended with 1% and 3% (w/w) SHC. The different lowercase letters represent significant difference between different treatments (Duncan’s multiple-comparison test, *p* < 0.05).

**Figure S4.** Enzyme activities of dehydrogenase (A), β-glucosidase (B), invertase (C) and cellobiohydrolase (D) of coastal salt-affected soils with or without char amendments. The values of qMB were calculated as follows: qMB = SOC decomposition rate/MBC. The different lowercase letters represent significant difference between different treatments (Duncan’s multiple-comparison test, *p* < 0.05).

**Figure S5.** Effect of char amendment on the original proportion of macroaggregates (A), microaggregates (B), silt-clay fractions (C) and aggregate stability index (D) of coastal salt-affected soils. Macroaggregates, microaggregates and silt-clay fractions indicated the soil aggregates with the size of 250–2000 μm, 53–250 μm and < 53 μm, respectively. Soil aggregate stability was indicated by values of mean weight diameter (MWD). CK: soil without char amendment; 1%SPC and 3%SPC: soils amended with 1% and 3% (w/w) SPC; 1%SHC and 3%SHC: soils amended with 1% and 3% (w/w) SHC. Error bars represent the standard deviation of the mean (*n* = 3). The different lowercase letters represent significant difference between different treatments (Duncan’s multiple-comparison test, *p* < 0.05).

**Figure S6.** Effect of char amendment on the calibrated proportion of macroaggregates (A), microaggregates (B) and silt-clay fractions (C) of coastal salt-affected soils. Macroaggregates, microaggregates and silt-clay fractions indicated soil aggregates with the size of 250–2000 μm, 53–250 μm and < 53 μm, respectively. CK: soil without char amendment; 1%SPC and 3%SPC: soils amended with 1% and 3% (w/w) SPC; 1%SHC and 3%SHC: soils amended with 1% and 3% (w/w) SHC. Error bars represent the standard deviation of the mean (*n* = 3). The different lowercase letters represent significant difference between different treatments (Duncan’s multiple-comparison test, *p* < 0.05).

**
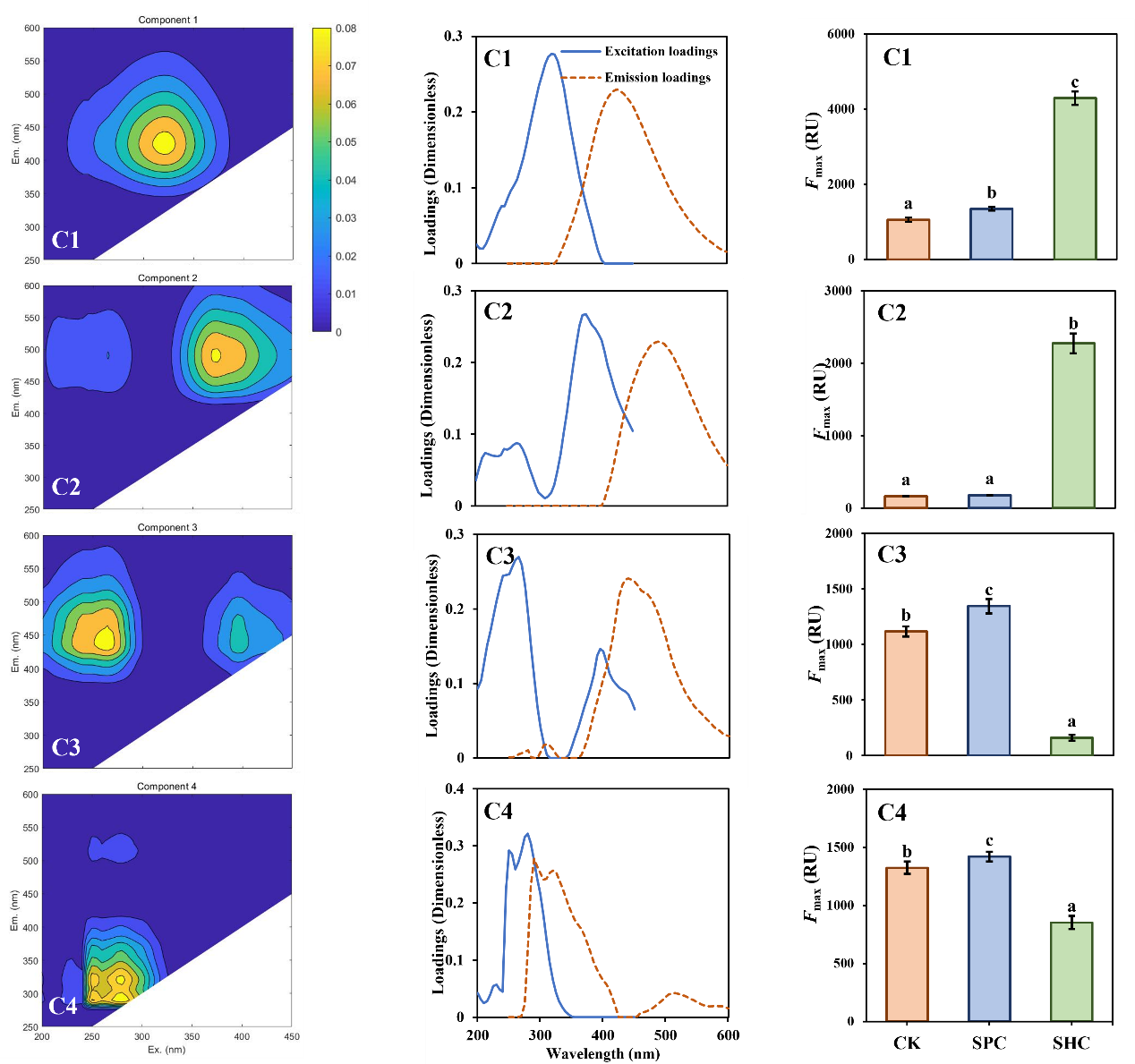
**

**(B)**

**(A)**

**Figure S7.** Effect of char amendments on the composition of soil dissolved organic matter, including contour plots of four components (A) and fluorescence spectra and intensity (B). Component 1 (C1, low molecular weight fulvic-acid substrates) (Ex/Em: 325/400 nm), component 2 (C2, phenolic, less aromatic structures and soluble microbial metabolic protein) (Ex/Em: 350/450 nm), component 3 (C3, humic acid-like substances consisting of phenols and other ingredients with aromatic structures) (Ex/Em: 250/440 nm) and component 4 (C4, aromatic protein substances tryptophan protein) (Ex/Em: 280/320 nm). Different letters indicate the significant differences between different treatments (Duncan’s multiple-comparison test, *p* < 0.05).

**Figure S8.** Three-dimensional fluorescence spectra of dissolved organic matter (DOM) extracted from control soil and char-treated soils. CK: soil without char amendment; 1%SPC and 3%SPC: soils amended with 1% and 3% (w/w) SPC; 1%SHC and 3%SHC: soils amended with 1% and 3% (w/w) SHC. Error bars represent the standard deviation of the mean (*n* = 3). Contour plots of four components: C1 (low molecular weight fulvic-acid substrates) (Ex/Em: 325/400 nm), C2 (phenolic, less aromatic structures and soluble microbial metabolic protein) (Ex/Em: 350/450 nm), C3 (humic acid-like substances consisting of phenols and other ingredients with aromatic structures) (Ex/Em: 250/440 nm) and C4 (aromatic protein substances tryptophan protein) (Ex/Em: 280/320 nm).

**
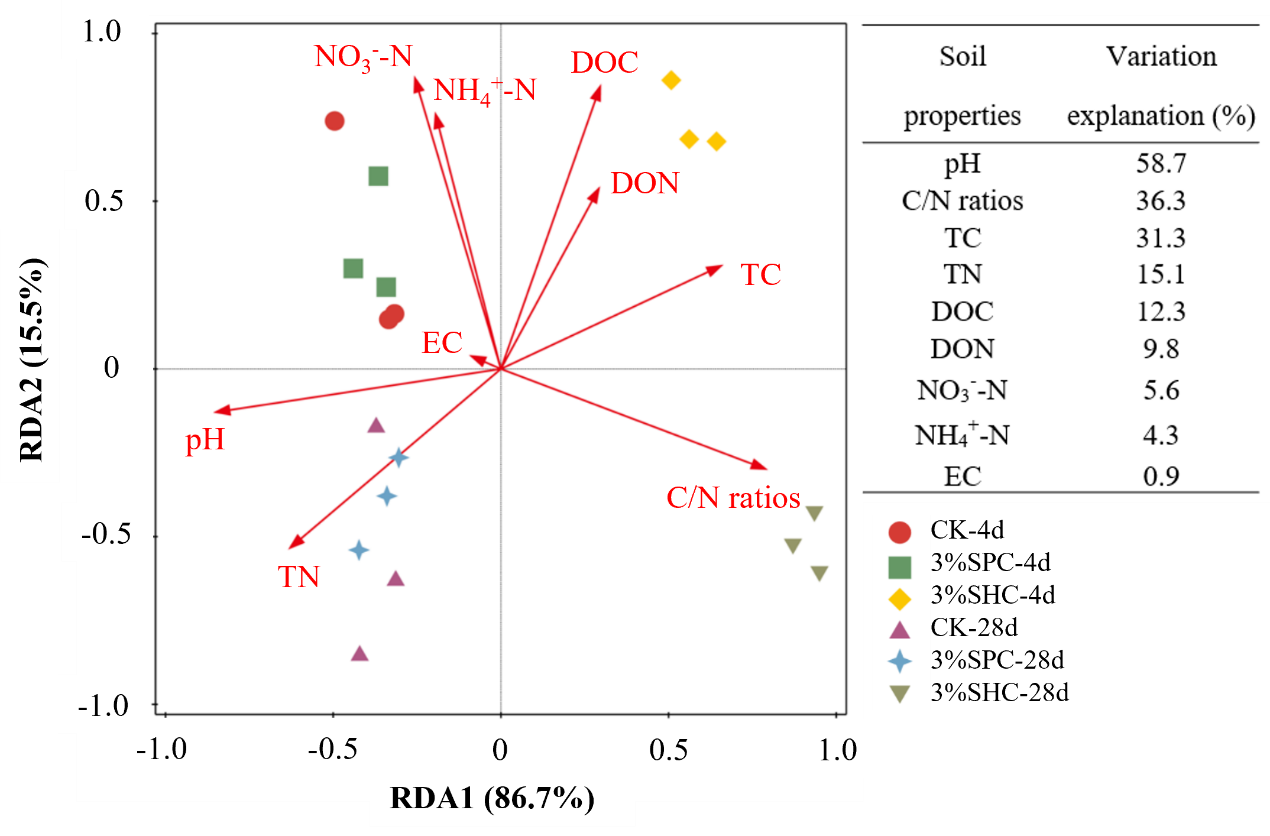
**

**Figure S9.** RDA analysis between bacterial community composition and soil properties. CK: soil without char amendment; 3%SPC and 3%SHC: soils amended with 3% (w/w) pyrochar and hydrochar, respectively.


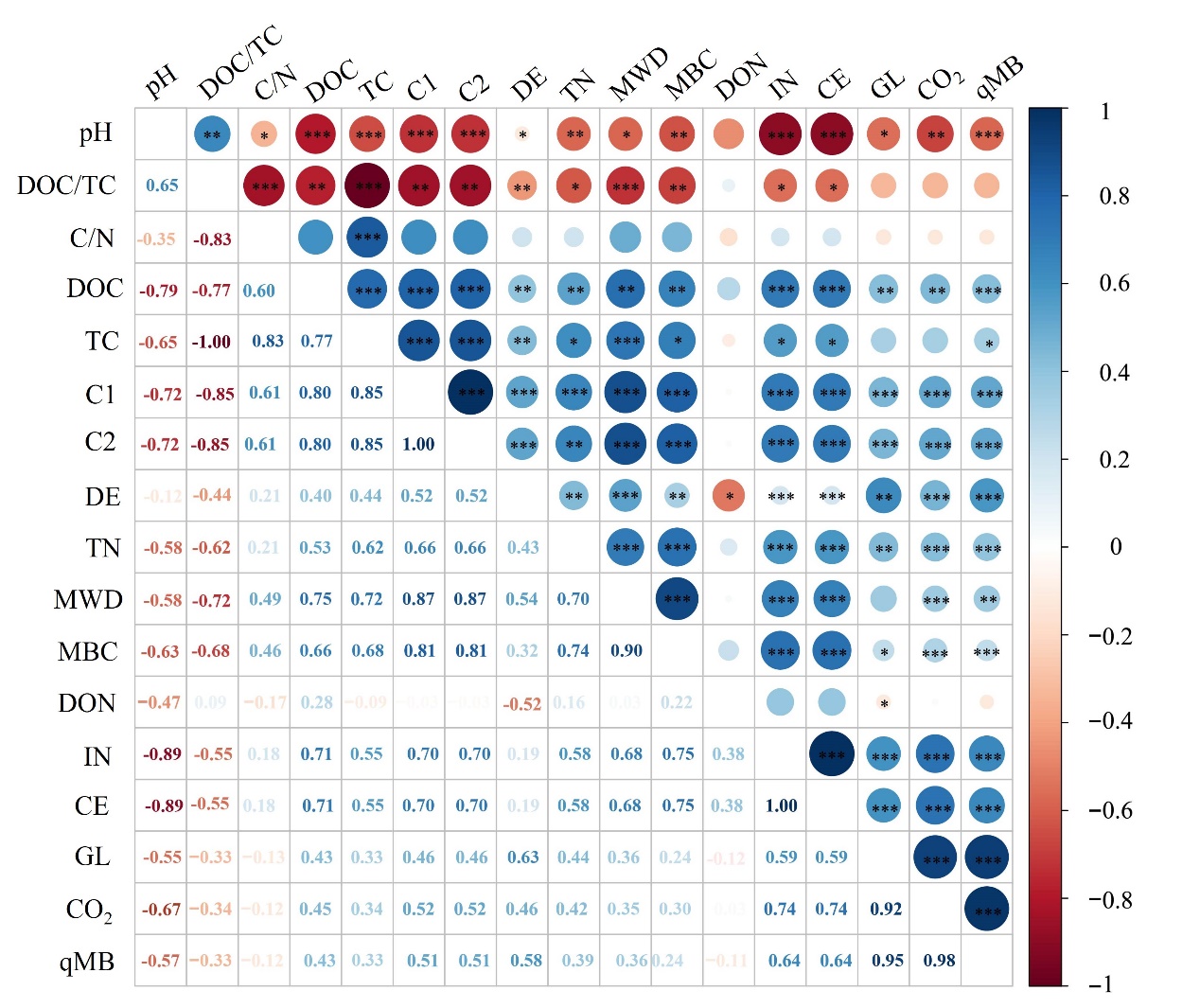


**Figure S10.** Spearman correlation analysis of soil CO_2_ emission, C-transforming enzyme activities and soil properties. DE: dehydrogenase, GL: β-glucosidase, IN: invertase, CE: cellobiohydrolase. *Correlation is significant at *p* < 0.05; **Correlation is significant at *p* < 0.01; *** Correlation is significant at *p* < 0.001.

**Reference**

1. You, Xiangwei, Xiao Wang, Ruixue Sun, Qiang Liu, Song Fang, Qingxian Kong, Xin Zhang, et al. 2023. “Hydrochar more effectively mitigated nitrous oxide emissions than pyrochar from a coastal soil of the Yellow River Delta, China.” *Science of The Total Environment* 858: 159628. https://doi.org/https://doi.org/10.1016/j.scitotenv.2022.159628

2. Zheng, H., X. Wang, X. Luo, Z. Wang, B. Xing. 2018. “Biochar-induced negative carbon mineralization priming effects in a coastal wetland soil: Roles of soil aggregation and microbial modulation.” *Science of The Total Environment* 610-611: 951-960. https://doi.org/10.1016/j.scitotenv.2017.08.166

3. Khosravi, Anahita, Hao Zheng, Qiang Liu, Masoud Hashemi, Yuanzhi Tang, Baoshan Xing. 2022. “Production and characterization of hydrochars and their application in soil improvement and environmental remediation.” *Chemical Engineering Journal* 430: 133142. https://doi.org/https://doi.org/10.1016/j.cej.2021.133142

4. Ni, Haowei, Xiaoyan Jing, Xian Xiao, Na Zhang, Xiaoyue Wang, Yueyu Sui, Bo Sun, Yuting Liang. 2021. “Microbial metabolism and necromass mediated fertilization effect on soil organic carbon after long-term community incubation in different climates.” *The ISME Journal* 15: 2561-2573. https://doi.org/10.1038/s41396-021-00950-w

5. Wei, Xiaomeng, Zhenke Zhu, Yi Liu, Yu Luo, Yangwu Deng, Xingliang Xu, Shoulong Liu, et al. 2020. “C:N:P stoichiometry regulates soil organic carbon mineralization and concomitant shifts in microbial community composition in paddy soil.” *Biology and Fertility of Soils* 56: 1093-1107. https://doi.org/10.1007/s00374-020-01468-7

6. Ma, Linna, Chaoxue Zhang, Xiaofeng Xu, Congwen Wang, Guofang Liu, Cunzhu Liang, Xiaoan Zuo, Chengjie Wang, Yixia Lv, Renzhong Wang. 2022. “Different facets of bacterial and fungal communities drive soil multifunctionality in grasslands spanning a 3500 km transect.” *Functional Ecology* 36: 3120-3133. https://doi.org/https://doi.org/10.1111/1365-2435.14220

7. Ashraf, M. N., M. A. Waqas, S. Rahman. 2022. “Microbial Metabolic Quotient is a Dynamic Indicator of Soil Health: Trends, Implications and Perspectives (Review).” *Eurasian Soil Science* 55: 1794-1803. https://doi.org/10.1134/S1064229322700119

8. Wang, Chao, Lingrui Qu, Liuming Yang, Dongwei Liu, Ember Morrissey, Renhui Miao, Ziping Liu, Qingkui Wang, Yunting Fang, Edith Bai. 2021. “Large-scale importance of microbial carbon use efficiency and necromass to soil organic carbon.” *Global Change Biology* 27: 2039-2048. https://doi.org/https://doi.org/10.1111/gcb.15550

9. Spohn, Marie, Karoline Klaus, Wolfgang Wanek, Andreas Richter. 2016. “Microbial carbon use efficiency and biomass turnover times depending on soil depth – Implications for carbon cycling.” *Soil Biology and Biochemistry* 96: 74-81. https://doi.org/https://doi.org/10.1016/j.soilbio.2016.01.016

10. Haj-Amor, Z., T. Araya, D. G. Kim, S. Bouri, J. Lee, W. Ghiloufi, Y. Yang, et al. 2022. “Soil salinity and its associated effects on soil microorganisms, greenhouse gas emissions, crop yield, biodiversity and desertification: A review.” *Science of The Total Environment* 843: 156946. https://doi.org/10.1016/j.scitotenv.2022.156946

11. Kumawat, Chiranjeev, Ajay Kumar, Jagdish Parshad, Shyam S. Sharma, Abhik Patra, Prerna Dogra, Govind K. Yadav, Sunil K. Dadhich, Rajhans Verma, Girdhari L. Kumawat. 2022. 'Microbial Diversity and Adaptation under Salt-Affected Soils: A Review', *Sustainability*.

12. Pokharel, Prem, Zilong Ma, Scott X. Chang. 2020. “Biochar increases soil microbial biomass with changes in extra- and intracellular enzyme activities: a global meta-analysis.” *Biochar* 2: 65-79. https://doi.org/10.1007/s42773-020-00039-1

13. Dorodnikov, Maxim, Evgenia Blagodatskaya, Sergey Blagodatsky, Sven Marhan, Andereas Fangmeier, Yakov Kuzyakov. 2009. “Stimulation of microbial extracellular enzyme activities by elevated CO_2_ depends on soil aggregate size.” *Global Change Biology* 15: 1603-1614. https://doi.org/https://doi.org/10.1111/j.1365-2486.2009.01844.x

14. Tian, Jing, Nianpeng He, Lauren Hale, Shuli Niu, Guirui Yu, Yuan Liu, Evgenia Blagodatskaya, Yakov Kuzyakov, Qun Gao, Jizhong Zhou. 2018. “Soil organic matter availability and climate drive latitudinal patterns in bacterial diversity from tropical to cold temperate forests.” *Functional Ecology* 32: 61-70. https://doi.org/https://doi.org/10.1111/1365-2435.12952

15. Haj-Amor, Zied, Tesfay Araya, Dong-Gill Kim, Salem Bouri, Jaehyun Lee, Wahida Ghiloufi, Yerang Yang, et al. 2022. “Soil salinity and its associated effects on soil microorganisms, greenhouse gas emissions, crop yield, biodiversity and desertification: A review.” *Science of The Total Environment* 843: 156946. https://doi.org/https://doi.org/10.1016/j.scitotenv.2022.156946

16. Zheng, X., X. Li, B. P. Singh, L. Wei, L. Huang, Y. Huang, Q. Huang, et al. 2021. “Biochar protects hydrophilic dissolved organic matter against mineralization and enhances its microbial carbon use efficiency.” *Science of The Total Environment* 795: 148793. https://doi.org/10.1016/j.scitotenv.2021.148793

17. Feng, Jiao, Dailin Yu, Robert L. Sinsabaugh, Daryl L. Moorhead, Mathias Neumann Andersen, Pete Smith, Yanting Song, et al. 2023. “Trade-offs in carbon-degrading enzyme activities limit long-term soil carbon sequestration with biochar addition.” *Biological Reviews* 98: 1184-1199. https://doi.org/https://doi.org/10.1111/brv.12949

18. McLeod, Morgan Luce, Lorinda Bullington, Cory C. Cleveland, Johannes Rousk, Ylva Lekberg. 2021. “Invasive plant-derived dissolved organic matter alters microbial communities and carbon cycling in soils.” *Soil Biology and Biochemistry* 156: 108191. https://doi.org/https://doi.org/10.1016/j.soilbio.2021.108191

19. Zimmerman, Andrew R., Lei Ouyang. 2019. “Priming of pyrogenic C (biochar) mineralization by dissolved organic matter and vice versa.” *Soil Biology and Biochemistry* 130: 105-112. https://doi.org/https://doi.org/10.1016/j.soilbio.2018.12.011

20. Sun, Yuqing, Xinni Xiong, Mingjing He, Zibo Xu, Deyi Hou, Weihua Zhang, Yong Sik Ok, Jörg Rinklebe, Linling Wang, Daniel C. W. Tsang. 2021. “Roles of biochar-derived dissolved organic matter in soil amendment and environmental remediation: A critical review.” *Chemical Engineering Journal* 424: 130387. https://doi.org/https://doi.org/10.1016/j.cej.2021.130387

21. Li, Ming, Afeng Zhang, Haiming Wu, Hai Liu, Jialong Lv. 2017. “Predicting potential release of dissolved organic matter from biochars derived from agricultural residues using fluorescence and ultraviolet absorbance.” *Journal of Hazardous Materials* 334: 86-92. https://doi.org/https://doi.org/10.1016/j.jhazmat.2017.03.064

22. Shao, Yuchao, Menggang Bao, Weizhong Huo, Rong Ye, Muhammad Ajmal, Wenjing Lu. 2023. “From biomass to humic acid: Is there an accelerated way to go?” *Chemical Engineering Journal* 452: 139172. https://doi.org/https://doi.org/10.1016/j.cej.2022.139172

23. Chen, Jiancheng, Hui Wang, Guoqing Hu, Xuhua Li, Yuanjie Dong, Yuping Zhuge, Hongbo He, Xudong Zhang. 2021. “Distinct accumulation of bacterial and fungal residues along a salinity gradient in coastal salt-affected soils.” *Soil Biology and Biochemistry* 158: https://doi.org/10.1016/j.soilbio.2021.108266

24. Sun, Ke, Lanfang Han, Yan Yang, Xinghui Xia, Zhifeng Yang, Fengchang Wu, Fangbai Li, Yanfang Feng, Baoshan Xing. 2020. “Application of Hydrochar Altered Soil Microbial Community Composition and the Molecular Structure of Native Soil Organic Carbon in a Paddy Soil.” *Environmental Science & Technology* 54: 2715-2725. https://doi.org/10.1021/acs.est.9b05864

25. Luo, Xianxiang, Leyun Wang, Guocheng Liu, Xiao Wang, Zhenyu Wang, Hao Zheng. 2016. “Effects of biochar on carbon mineralization of coastal wetland soils in the Yellow River Delta, China.” *Ecological Engineering* 94: 329-336. https://doi.org/10.1016/j.ecoleng.2016.06.004

26. Zheng, Hao, Nianlin Feng, Tianning Yang, Mei Shi, Xiao Wang, Qian Zhang, Jian Zhao, Fengmin Li, Ke Sun, Baoshan Xing. 2021. “Individual and combined applications of biochar and pyroligneous acid mitigate dissemination of antibiotic resistance genes in agricultural soil.” *Science of The Total Environment* 796: 148962. https://doi.org/https://doi.org/10.1016/j.scitotenv.2021.148962

27. Zheng, Hao, Xiao Wang, Xianxiang Luo, Zhenyu Wang, Baoshan Xing. 2018. “Biochar-induced negative carbon mineralization priming effects in a coastal wetland soil: Roles of soil aggregation and microbial modulation.” *Science of the Total Environment* 610–611: 951-960. https://doi.org/10.1016/j.scitotenv.2017.08.166

28. Li, Detian, Huiting Li, Danyan Chen, Lihong Xue, Huayong He, Yanfang Feng, Yang Ji, Linzhang Yang, Qingnan Chu. 2021. “Clay-hydrochar composites mitigated CH_4_ and N_2_O emissions from paddy soil: A whole rice growth period investigation.” *Science of The Total Environment* 780: 146532. https://doi.org/https://doi.org/10.1016/j.scitotenv.2021.146532

29. Zheng, Hao, Xiao Wang, Xianxiang Luo, Zhenyu Wang, Baoshan Xing. 2018. “Biochar-induced negative carbon mineralization priming effects in a coastal wetland soil: Roles of soil aggregation and microbial modulation.” *Science of The Total Environment* 610-611: 951-960. https://doi.org/https://doi.org/10.1016/j.scitotenv.2017.08.166

30. Jia, L., E. Gou, H. Liu, S. Lu, S. Wu, H. Wu. 2019. “Exploring Utilization of Recycled Agricultural Biomass in Constructed Wetlands: Characterization of the Driving Force for High-Rate Nitrogen Removal.” *Environmental Science & Technology* 53: 1258-1268. https://doi.org/10.1021/acs.est.8b04871

31. Mostofa, Khan M. G., Yuan Jie, Hiroshi Sakugawa, Cong-Qiang Liu. 2018. “Equal Treatment of Different EEM Data on PARAFAC Modeling Produces Artifact Fluorescent Components That Have Misleading Biogeochemical Consequences.” *Environmental Science & Technology* 53: 561-563. https://doi.org/10.1021/acs.est.8b06647

32. Liu, Yong-Xin, Lei Chen, Tengfei Ma, Xiaofang Li, Maosheng Zheng, Xin Zhou, Liang Chen, et al. 2023. “EasyAmplicon: An easy-to-use, open-source, reproducible, and community-based pipeline for amplicon data analysis in microbiome research.” *iMeta* 2: e83. https://doi.org/https://doi.org/10.1002/imt2.83

33. Chen, D., X. Gu, W. Zhu, S. He, F. Wu, J. Huang, W. Zhou. 2019. “Denitrification- and anammox-dominant simultaneous nitrification, anammox and denitrification (SNAD) process in subsurface flow constructed wetlands.” *Bioresource Technology* 271: 298-305. https://doi.org/10.1016/j.biortech.2018.09.123

34. Hernandez, D. J., A. S. David, E. S. Menges, C. A. Searcy, M. E. Afkhami. 2021. “Environmental stress destabilizes microbial networks.” *The ISME Journal* 15: 1722-1734. https://doi.org/10.1038/s41396-020-00882-x

35. Wen, Tao, Penghao Xie, Shengdie Yang, Guoqing Niu, Xiaoyu Liu, Zhexu Ding, Chao Xue, Yong-Xin Liu, Qirong Shen, Jun Yuan. 2022. “ggClusterNet: An R package for microbiome network analysis and modularity-based multiple network layouts.” *iMeta* 1: e32. https://doi.org/https://doi.org/10.1002/imt2.32
